# Food Sovereignty of the Indigenous Peoples in the Arctic Zone of Western Siberia: Response to COVID-19 Pandemic

**DOI:** 10.3390/ijerph17207570

**Published:** 2020-10-18

**Authors:** Elena Bogdanova, Sergei Andronov, Ildiko Asztalos Morell, Kamrul Hossain, Dele Raheem, Praskovia Filant, Andrey Lobanov

**Affiliations:** 1 Department of Economics and Management, Northern Arctic Federal University, 164500 Arkhangelsk, Russia; 2 National Medical Research Center for Rehabilitation and Balneology, Ministry of Health of the Russia, 121099 Moscow, Russia; sergius198010@mail.ru (S.A.); alobanov89@gmail.com (A.L.); 3 Department of Urban and Rural Development, Swedish University of Agricultural Sciences, 75007 Uppsala, Sweden; ildiko.asztalos.morell@slu.se; 4 Northern Institute of Environmental and Minority Law, Arctic Center of the University of Lapland, 96101 Rovaniemi, Finland; kamrul.hossain@ulapland.fi; 5 Association of Reindeer Herders in YNAO, 629000 Salekhard, Russia; filant76@mail.ru

**Keywords:** food sovereignty, reindeer herding, food value chain, Indigenous peoples, COVID-19 pandemic, the Arctic, Western Siberia, Yamal-Nenets Autonomous Okrug

## Abstract

This article presents the challenges facing reindeer herding as being both a profitable business and part of the traditional culture of the nomadic Indigenous peoples in the Arctic zone of Western Siberia which addresses substantial needs of the local population. Reindeer herding products are used as traditional nutrition, and as effective preventive means and remedies for adapting to the cold and geomagnetic activity in the High North. Export trends of traditional reindeer products have decreased local Indigenous peoples’ access to venison and had a negative impact on their health. Due to the COVID-19 pandemic, it is especially urgent for the Indigenous peoples to have sufficient access to traditional food and be involved in policy decision-making to maintain this traditional business. We aim to analyze the dependencies of Indigenous peoples on the reindeer produce–exporting “food value chain” and explore how (1) the independence of reindeer herders could be increased in these export chains and (2) how provision of their products to local communities could be secured. The study takes a multidisciplinary approach based on policy and socioeconomic analyses with input from medical research. Primary sources include data collected from interviews and surveys of Indigenous peoples during expeditions to the Nyda settlement, the Nydinskaya tundra, the Tazovsky settlement, the Tazovskaya tundra, the Nakhodka tundra, the Gyda and Gydansky settlements, the Yavai-Salinskaya tundra, the Seyakha settlement, the Seyakhinskaya and Tambeyskaya tundras located along the southern coast of the Ob Bay, the northeast coast of the Yamal Peninsula, the Tazovsky and Gydansky Peninsulas, and the Shuryshkarsky district. Data were collected during the summers and winters of 2014–2020.

## 1. Introduction

Reindeer herding is deeply rooted in the traditional food culture of the Indigenous peoples in the Arctic zone of Western Siberia. Strengthening the position of reindeer herders in the food value chain could contribute to securing healthy, accessible, and culturally appropriate food for local communities and reindeer herders, while promoting the desired lifestyle of the given group. Food security for this group of Indigenous peoples cannot be addressed in isolation, but is linked to their economic security and food sovereignty. Therefore, both physical and economic access to reindeer meat in sufficient quantity is connected to their engagement in the export market and their participation in the food value chain. However, due to export trends in reindeer products, the needs and position of Indigenous peoples in the food value chain are jeopardized. Additionally, COVID-19 has brought yet other stressor for this group. As a result, the question of food sovereignty has emerged in evaluating how these people experience challenges to sovereignty over their own food system. Food sovereignty generally refers to a situation where local and Indigenous peoples possess the necessary control over the whole process of a food system. It is about “peoples’ right to define their own policies and strategies for the sustainable production, distribution and consumption of food that guarantees the right to food for the entire population, on the basis of small- and medium-sized production, respecting their own cultures and the diversity of peasant, fishing and indigenous forms of agricultural production, marketing and management of rural areas, in which women play a fundamental role” (World Forum on Food Sovereignty, 2001) [1].

The Six Pillars of Food Sovereignty, developed by Nyéléni, 2007 (Food Secure Canada, 2012) [2], places people’s need for food at the center of policy, and insists that food is more than just a commodity. Policy should be in line with “supporting sustainable livelihoods”, “reducing the distance between suppliers and consumers”, “placing control in the hands of local food suppliers”, “recognizing the need to inhabit and share territories,” building commodity production on traditional knowledge”, and “maximizing the contributions of ecosystems” [2]. It is also strongly linked to food security related to food independence, the physical and economic accessibility of food, and the safety of available food (Rome Declaration on World Food Security and the World Food Summit Action Plan, 1996; Declaration of the World Summit on Food Security, 2009; Doctrine of Food Security of the Russian Federation, 2010).

The concept of food sovereignty challenges global food markets and empowers local actors [3]. It has special implications for Indigenous communities [4]. Taking the conditions of First Nation Indigenous people as a comparative point of analysis, Desmarais and Wittman (2014) identify that alarming problems of ill-health are closely related to food insecurity, which is in turn related to the decline of traditional food systems [5]. Among the reasons for this decline, the most important are disrupted access to land, traditional food, food trading and knowledge networks. Thus, traditional food, health and culture are tightly interconnected. The British Columbia Working Group on Indigenous Food Sovereignty defined the needs of Indigenous peoples to form sovereignty rights and the power of each nation in identifying the characteristics of their culture, as well as ways to enable Indigenous communities to “sustain traditional hunting, fishing, gathering, farming and distribution practices” [5]. Among the principles of culture, the most important element is the sacred nature of food: “Food sovereignty understands food as sacred, part of the web of relations with the natural world that define culture and community” (People’s Food Policy Project, 2011) [6]. Thus, the sacred nature of food implies that “it cannot be treated as a commodity, manipulated into junk foods or taken from people’s mouths to feed animals or vehicles” [7]. In the traditional food systems of Indigenous societies, reindeer form part of the culture and practices, with sacred and spiritual connotations [8]. The health of reindeer, nature and humans are tightly interrelated. So, a reindeer is utilized in a holistic manner. In contrast, when reindeer become goods on the free market, they are turned into a commodity, where utilization of body parts is compartmentalized.

Indigenous health and adaptation to the harsh conditions of the Arctic depend on the consumption of traditional products (local fish, reindeer meat, reindeer liver and blood, and wild plants) [9]. Reindeer meat has a specific health-promoting composition, and so is a necessary substance for maintaining a healthy diet in circumpolar circumstances and for decreasing the risk of chronic diseases [10,11,12,13,14]. It is the main tool for preventing cardiovascular and respiratory diseases, as well as metabolic disorders for the Indigenous peoples [15]. Dietary changes result in the declining health of the Indigenous population [16]. With the loss of traditional nutrition, hypertension, dyslipidemia, chronic bronchitis and obesity become even more prevalent problems among Indigenous peoples [17]. Maintenance of a traditional diet is closely related to the maintenance of the traditional food system, which is tightly interwoven with the culturally, socially and environmentally embedded practices of reindeer herding. Indigenous peoples and their traditional food systems emerged in harmony with nature and contain knowhow on the sustainable use of natural resources in ways that contribute to their health. New stressors related to the incorporation of reindeer exports in global markets force adjustments to the demands and infrastructure of markets. This implies the danger of new threats to the continued practice of environmentally, socially and economically sustainable Indigenous reindeer herding practices [12,18].

In meeting the nutritional needs of the Indigenous peoples in the Russian Arctic, it is vital that a major source of nutrition such as reindeer meat, which is readily available in the community, is well supported by all stakeholders. Reindeer meat offers a rich source of protein, minerals, and essential fatty acids, and is culturally acceptable in these Arctic communities [3]. Reindeer have contributed to the standard of living in these communities and served as a source of income from the sale of reindeer products (skin, meat, bones, velvet antlers, blood, etc.) which contribute to the value chain. The marketing of these products helps to create jobs and improve purchasing power for the suppliers, which makes other food products affordable. Food sovereignty emphasizes the promotion of small- and medium-sized production with particular respect for Indigenous cultures and traditional forms of agricultural production, management of land use and marketing [19]. The successful entrepreneurship of value addition in enhancing food sovereignty amongst the Indigenous peoples in the Russian Arctic zone of Western Siberia depends on collaboration amongst stakeholders. Recent trends of integration of reindeer herding producers from Western Siberia into global value chains have complemented national and Indigenous economies. However, increased exports from the area adversely impact the meat available locally and undermines the health of the Indigenous peoples and local communities. Challenges faced by most reindeer herders during the COVID-19 pandemic have accentuated these vulnerabilities.

In this study, we focus on integrating the different aspects of the reindeer food value chain in a multi-disciplinary approach to strengthen the food sovereignty of the Indigenous peoples in the Arctic zone of Western Siberia, and reflect on the main challenges of the COVID-19 pandemic.

## 2. Materials and Methods

### 2.1. Setting: The Yamal-Nenets Autonomous Okrug (YNAO): Geographic, Population and Ethnic Structure

**Figure 1:**
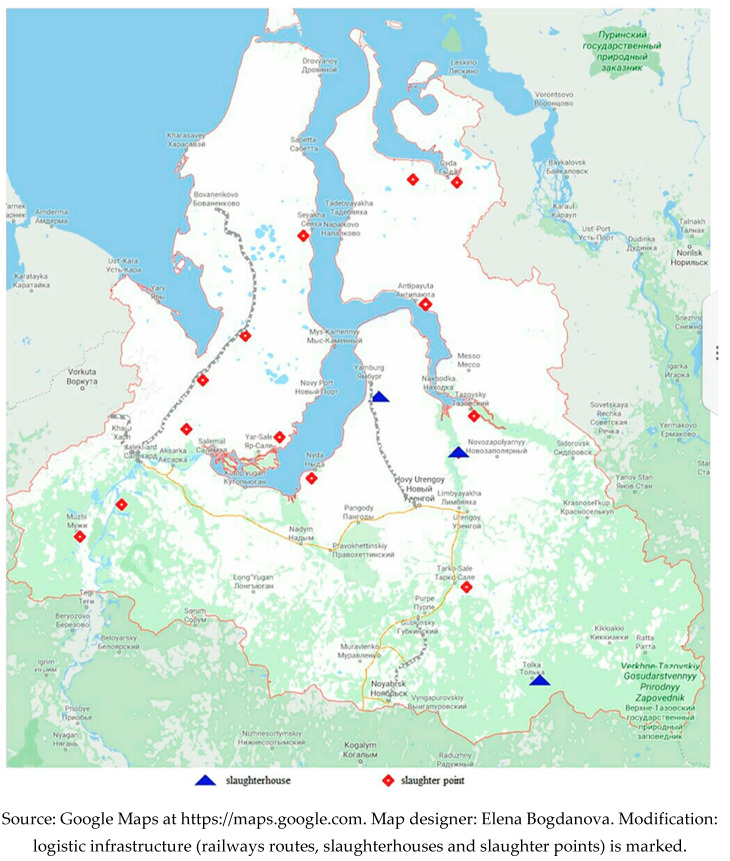
The territory of the Yamal-Nenets Autonomous Okrug.

YNAO, the geographic focus of our research, is an important region for the Indigenous peoples of Russia and is located in the circumpolar northwest of West Siberia (Figure 1). It has a population of 544,008 people [20] living in an area of 769,250 square kilometers [21]. The population density is 0.71 people per square kilometer. The location of YNAO significantly impacts the traditional occupations in this region (reindeer herding, hunting, fishery, etc.) as more than half of its territory is located beyond the Arctic Circle. It is a unique territory because almost half of the minority Indigenous population of the Russian Arctic (about 45,000 people) resides there, including the Nenets, the Khanty, the Selkups and the Komi-Zyryans [22]. Nearly half of the Indigenous residents are still nomadic. The culture, health and social well-being of Indigenous peoples are strongly linked to a traditional lifestyle that is the basis for meeting their vital needs and helping them survive in the severe Arctic areas.

### 2.2. Study Design

In this paper, we present the results of a quantitative and qualitative analysis of food sovereignty in reindeer herding communities living and practicing nomadism in the remote territories of the Yamal-Nenets Autonomous Okrug. We aimed to analyze the dependencies of Indigenous peoples on the reindeer produce exporting “food value chain” and to explore how the independence of reindeer herders could be increased in these export chains and how the provision of their products to local communities could be secured, while also addressing the challenges posed by the COVID-19 pandemic. 

### 2.3. Measurement Tools and Methodology

This study takes a multidisciplinary approach based on policy and socioeconomic analyses with input from medical research. The primary sources include data collected from interviews and surveys of Indigenous peoples during expeditions to the Nyda settlement, the Nydinskaya tundra, the Tazovsky settlement, the Tazovskaya tundra, the Nakhodka tundra, the Gyda and Gydansky settlements, the Yavai-Salinskaya tundra, the Seyakha settlement, the Seyakhinskaya and Tambeyskaya tundras located along the southern coast of the Ob Bay, the northeast coast of the Yamal Peninsula, the Tazovsky and Gydansky Peninsulas, and the Shuryshkarsky district. Data were collected in the summers and winters of 2014–2020. Fieldwork was conducted by researchers of the YNAO Arctic Scientific Research Centre, the National Medical Research Center for Rehabilitation and Balneology, the Northern Arctic Federal University and the Association of Reindeer Herders in YNAO (two of the researchers were Indigenous). Secondary sources used in the study consist of official information requested from local authorities, public statistical data and official government reports.

The methodology of the study is based on FAO’s (Food and Agriculture Organization of the United Nations) sustainable food value chain (FVC) framework. The core FVC comprises the value chain actors who produce or procure products from the upstream level, add value to these products and then sell them on to the next level. These actors carry out four functions: production, aggregation, processing, and distribution (wholesale and retail). FVC actors are linked to each other and their wider operating environment through a governance structure [23]. Following this approach, we presented the food value chain as a network of stakeholders that includes those involved in reindeer herding husbandry (producers), people responsible for purchasing, storage, sanitary control and transportation of raw reindeer products to producers (brokers), processing (processors), and exporting, distributing and selling reindeer herding products (distributors) to consumers who shop for and consume this food. Also included as stakeholders are government, non-governmental organizations (NGOs), and regulators that monitor and regulate the entire reindeer food value chain from producers to consumers.

While collecting the information from local authorities, public statistical data and official government reports, we implemented quantitative methods of analysis to collect data on the number of different producers of reindeer products, the scale of reindeer livestock, and the results of slaughter campaigns and sale to local communities as well as national and international markets.

The data received from the semi-structured interviews showed the ways reindeer products are utilized and revealed the key issues of individual reindeer herders as stakeholders in the FVC, as well as the challenges raised by the COVID-19 pandemic.

Statistical analyses were performed using Microsoft Excel 2016 and SPSS Statistics 23.0 (IBM, Saint Petersburg, Russia). Significant differences were defined at a *p*-value <0.05.

### 2.4. Study Population

The participants in the study were asked to fill out the questionnaire and interviewed while undergoing a medical examination conducted by the YNAO Arctic Scientific Research Centre and the National Medical Research Center for Rehabilitation and Balneology at health care institutions—municipal hospitals and feldsher-midwife medical stations in remote settlements. The participants were also recruited during the fieldwork in summer 2020 to study the impact of the COVID-19 pandemic on reindeer herding activities. The inclusion criteria for the respondents were: aged over 18, of Indigenous origin, Indigenous language speaker, involved in reindeer herding, a nomadic or semi-nomadic lifestyle, and residing in the tundra or in the settlements of the Arctic zone of Western Siberia for over five years. The participants received information about the program both verbally and in writing. They also provided written informed consent. The consent form stated that participation was voluntary and assured the confidentiality of the participants, whose personal data were anonymized, numbered, and added to de-identified databases. The interviews were recorded and also included in the database.

### 2.5. Ethics Approval

The study was approved by the Ethics Committee of the Arctic Scientific Research Centre of Yamal-Nenets Autonomous Okrug, Salekhard, Russian Federation, on 18 January 2013 (approval protocol No. 01/1-13).

## 3. Results and Discussion

Two hundred and sixty-five semi-structured interviews were conducted based on the interview guide developed and approved by the Northern Arctic Federal University and YNAO Arctic Scientific Research Centre. Two hundred and fifty-two surveys with fixed questions were received. 

Reindeer husbandry is the leading ethno-forming branch of the agro-industrial complex in the region. YNAO has the highest number of domesticated reindeer herds in the world, which has made it a prosperous reindeer herding area [24,25] with sustainable nomadic reindeer herding husbandry [26]. However, over the past two years, reindeer livestock has decreased by 11.3% from 2015 numbers (2015 numbers: 733; 2016: 465; 2017: 788; 2018: 690; 2019: 650) [27]. 

In YNAO, the network of stakeholders involved in producing, slaughtering, processing, distributing and consuming reindeer products forms the food value chain that is presented in Table 1.

The producers of reindeer products in YNAO (different types of reindeer herding enterprises, “national communities” of the Indigenous peoples, peasant farms and individual reindeer herders) provide reindeer husbandry and trade food commodities, such as raw reindeer products (venison, blood, fat, skin, camus, antlers, velvet antlers, bones, and other by-products). In 2020, in YNAO, there were 19 agricultural enterprises, 447 national communities (obschina), 11 peasant farms specializing in reindeer herding and processing reindeer products, and 2839 individual reindeer herders (Table 2). 

Collective reindeer herding in YNAO (agricultural reindeer herding enterprises, national communities and peasantry farms) is an effective organizational form for the Indigenous economy involved in producing, slaughtering and processing reindeer products. It is rooted in both the Soviet period (a state farm or sovkhoz) and traditional forms of Indigenous peoples’ cooperation (national community or obschina [29,30], and peasantry farms [31,32] as joint family businesses). Anthropologist V. Vladimirova noticed that the cooperative organization of reindeer husbandry in the Russian North reproduces the economic and social patterns that were developed during the Soviet period, also adapting and incorporating elements of traditional indigenous social orders. “Such social arrangements and the accompanying moral values are embedded in the reindeer herding economy, and it is their persistence that Indigenous people achieve through adhering to cooperative values” [33]. This mixed culture-based approach to the collective organization of reindeer farming still provides sustainability for the sovkhoz in Western Siberia. Strong dependence of reindeer herding husbandry on the Indigenous traditional lifestyle and “democratic leadership” [34,35] also maintain such traditional forms of Indigenous peoples’ self-organization in YNAO as a “national community” (obschina), a collaborative economy of family, clan and territorial neighbors that protect and maintain their traditional lifestyle, economic activities and culture [29]. 

The recognition of different forms of reindeer herding differs depending on the state. Indigenous reindeer herders’ communities, unlike agricultural enterprises, are not strongly subsidized by the regional budget (apart from for producing reindeer meat). So some reindeer herders find transforming a national community into a peasantry farm or an agricultural production cooperative attractive because of access to government support, subsidies and future prospects for developing business. Bogdan O., a reindeer herder from the Laborovskaya tundra, thinks that “The head of a family with a reindeer herd will be able to register a peasant farm as an enterprise in the future if he wants to enlarge and offer new jobs to unite the farms of his relatives or neighbors into a cooperative.” 

However, individual reindeer herders are still the main producers of reindeer products in YNAO (60%) (see Table 1). They still prefer not to join any collective reindeer organizations and operate reindeer herding as individuals with their families—accompanied in the tundra by parents, spouses and children, and sometimes brothers, sisters, uncles and aunts. They are also cautious about any changes and paperwork. For example, the reindeer herder Ilia V. from the Laborovskaya tundra explains: *“Joining peasant farms or entering a community to receive grants and subsidies? It is difficult because I cannot make documents and reports for grants well. My family and I are nomads in the tundra with my reindeer far away in the Karskoe Sea. I do not have time to go to the authorities and do not know how to run this business and what papers are needed. I finished seven years of formal schooling and escaped to the tundra. Reindeer herding is my life now.”* This reveals that reindeer herding husbandry in Western Siberia is still mostly focused on the subsistence economy combined with commodity production. In extreme climate conditions, societies do not use the revolutionary achievements of the Neolithic period: they do not strive to switch to a productive type of agriculture [36], since a herder interacts with the environment as a partner who feels like an equal part of it [37].

In the Arctic, most Indigenous communities belong to a mixed “subsistence-cash” economy [38]. In YNAO, individual reindeer herders, as part of Arctic communities, represent two types of social economy: commodity production—producing and sharing for sales to generate profit—and subsistence production—meeting the needs of reindeer herders and their families. The results of our research showed that the reindeer herders of the Tazovskaya, Messoyakhinskaya, Antipayutinskaya, the Tanamskaya tundras of the Tazovsky district, the Yamal and Gydan Peninsulas, and the Yamal’sky district of YNAO are mainly integrated into the commodity production of meat and velvet antlers thanks to relatively good logistics and presence of slaughterhouses. Subsistence farming, focused on providing a family with food and clothing, prevails in the northern part of the Gydan Peninsula, on the coast of the Yuratskaya Bay and other parts of the Tazovsky district, which are logistically remote from settlements, slaughterhouses and large oil and gas deposits. In the Priural’sky district, there is both commodity and subsistence reindeer production because of developed logistic complexes (Figure 1).

Reindeer herding is impacted annually by climate change [39,40,41,42], overgrazing of reindeer pastures [43,44], the growing cost of living in the tundra and changing government regulations for reindeer herding as a traditional occupation for Indigenous peoples. In 2020, the reindeer herding economy in YNAO has also been strongly affected by the COVID-19 pandemic since individual reindeer herders, as the main producers, are a vulnerable group faced with a number of issues due to: (1) limited access to food, fuel, medications, vaccination procedures for reindeer and slaughtering facilities; (2) increased production costs; and (3) dropping prices for reindeer products. This falls in line with global trends: the pandemic has impacted food security and food sovereignty, which are “greatly affected due to mobility restrictions, reduced purchasing power, and with a greater impact on the most vulnerable population groups” [45]. 

Reindeer herding in the Russian Arctic is mostly a subsidized business because of high productivity costs and low incomes. This makes the impact of COVID-19 very serious for reindeer herders who perceive the coronavirus, icing and anthrax as evil forces that “were fabricated to reduce the number of people in the tundra” [46]. It has made reindeer herders change their nomadic schedule and completely limited nomadic people’s access to the settlements and cities where they can buy food, fuel and medications for reindeer. Alena V., a reindeer herder’s wife, complained: “We usually buy products in April in Labytnangi at the base or ‘from sledges.’ But this year, due to the pandemic, my family did not have time to buy fuel, essential goods, food, or tarpaulin. We had to start the summer nomadic period earlier – in early April – and change our route. We bought food and went to the tundra with three other reindeer herding families.” Other reindeer herding families made the decision to stay closer to the settlement during the period of pandemic limitations. For example, Venera S. noticed: “It is expensive to come to the city from the tundra—16,000 (*100 Russian Rubles = 1.12 € or 1.32 US$.) rubles one way. One also needs to buy food and live somewhere in the city. The reindeer stay with our relatives during this time. Our family was supposed to nomad during spring and summer this year, but because of the pandemic, it was impossible to go anywhere, and our family made the decision to stay close to the settlement”.

Limited access to vaccines for reindeer during the COVID-19 pandemic has become a sensitive issue for food security. Most vaccination procedures are subsidized by the local government of YNAO to support reindeer herding and control the pandemic. However, in spring (from mid-February to April) only 40% of about 220,000 reindeer that were planned to be vaccinated against anthrax received a vaccine from the veterinary service of YNAO. The summer vaccination period in the Priural’sky, Yamal’sky, Tazovsky and Purovsky districts of YNAO was delayed and started in late June [47]. Timely preventive vaccination and treatment (against anthrax, salmonellosis, trichophytosis, colibacophytosis, brucellosis, erysipelas, classical swine flu, plague, rabies, edemagenosis, helminthiasis, etc.) are important for maintaining reindeer health and addressing the restrictions on slaughter since reindeer herders are not allowed to slaughter animals without a vaccination [48] and cannot get government donations for murrain [49]. Besides, some diseases result in production losses (loss of weight of a reindeer [28]) followed by decreased profit for reindeer herders at the slaughtery.

The medication for some other reindeer diseases (i.e., necrobacillosis) is not subsidized by the government. Thus, in summer 2020, during the period of limitations due to the COVID-19 pandemic, most nomadic reindeer herders suffered greatly because they could not get access to pharmacies in the cities and had to buy anti-necrobacillosis medications at the trading posts (faktoria) in remote areas and pay unfair prices. Viktoria Z., a reindeer herder from Aksarka, said: “It was very difficult to get medical supplies for reindeer during the summer period. Because of the coronavirus, it was impossible to buy them in pharmacies, and we were not given the medicine that reindeer really need when they limp—antibiotics. (…) Now we have not received any medications from the veterinary service. It used to be good: first-aid kits were given to us. Now, the veterinary service has only supplied us with a vaccine against gadflies.” Bogdan A., the reindeer herder from the Laborovskaya tundra, noticed: “During the summer quarantine, <…> I had to buy medicines for reindeer that were five times more expensive at the trading post. I paid 20,000 rubles for it.” Moreover, reindeer herders sometimes could not find necessary products at the trade posts. Ilia S., the reindeer herder from the Laborovskaya tundra, recounted: *“On the way, we passed trading posts. Their prices were too high and the shops were almost empty.”* Some other reindeer herders asked their relatives and friends in the city to buy and send necessary medications. Anna I., a reindeer herder from Aksarka, recalled: “I asked someone in Salekhard and kind people helped me get medications. Now I order them from Ekaterinburg.” Challenges of delayed vaccination in the spring and summer could negatively impact the results of the slaughter campaign from November 2020 to January 2021 due to the possibility of decreased reindeer health and livestock approved for slaughter, and could make the reindeer herding business even less profitable for individuals.

High production costs were also a result of limited access to petroleum stations during the COVID-19 pandemic. Most individual reindeer herders had to cooperate and share logistical costs of hiring a taxi to get to petroleum stations close to the city and back to the tundra. However, this still increased their expenses for fuel by almost 50%. Mikhail V., a reindeer herder from the Priural’sky district, explained: “It was very difficult for us to get the fuel, which reindeer herders need most of all. Our petroleum station in Aksarka was also closed, so we had to go to Salekhard city and hire a taxi for 3000 rubles one way. If two or three people split the cost, then it was possible to buy more barrels of fuel since the cost was less. However, if only one reindeer herder was going, it was a very high price to pay.” In YNAO, reindeer herding as commodity production strongly depends on the price of fuel and localization of logistic centers and slaughter facilities. The interviews of reindeer herders in the Tazovsky district of YNAO (n = 84) showed that the price of the delivery of fuel varied depending on distance from regional centers and petroleum stations. For example, in the Tazovsky settlement, the cost of petroleum AI-92 was 43 rubles per liter, in the trade point (faktoria) of Yuribei Gydansky, 94 rubles, in the Gyda settlement, 109 rubles, and on Oleniy Island, 138 rubles. Thus, the logistical expenses of reindeer herders increased in the remote areas and made them change their nomadic routes to pass by slaughter facilities or collaborate with other types of broker (i.e., local merchants).

Processors are supplied with reindeer products directly from the reindeer herding enterprises or via brokers who purchase them from individual reindeer herders or collective reindeer herders’ farms, giving them a choice to sell their products to: (1) slaughterhouses; (2) state farms (sovkhoz); (3) local trade points (faktoria); or (4) local merchants in the tundra. These brokers have to take responsibility for sanitary (veterinary) control, appropriate storage and delivery of reindeer products to processors, and offer different terms of sale for reindeer herders who are also producers. As a result of the slaughter campaign in 2019, 1847 tons of reindeer meat were harvested (Department of Agro-industrial Complex of YNAO, 2020). 

In YNAO, there are 13 slaughter complexes and three slaughter points (Figure 1) near logistical infrastructure—railway or marine logistical routes. In 2019, they slaughtered in total 46,033 reindeer (1643 tons of venison), of which 26,259 (797.5 tons) came from agricultural enterprises, 16,281 (695.6 tons) from “national communities,” 1662 (70.8 tons) from peasant farms, and 1831 (79 tons) from individual reindeer herders [27]. In 2020, 85% of the interviewed individual reindeer herders had faced some issues due to the COVID-19 pandemic: (1) slaughterhouses sorted out the meat in the most profitable manner to decrease the price; (2) a live reindeer was received by a slaughterhouse but reindeer herders were paid only for the meat, and they had to buy the skins and camuses if they wanted them back or accept the decreased price for the reindeer meat (category II instead of category I); and (3) reindeer herders had to wait up to three months to be paid for slaughtered meat. Sergei S., a reindeer herder, complained about the delayed payment for his products and an unfair deal with a slaughterhouse: “This year, we slaughtered meat at Antipayuta. If we slaughtered a lot of reindeer (more than 100 heads), the money was not paid immediately, but if we slaughtered much less reindeer, then the money was paid immediately. We delivered about a ton of meat and had to wait for the money for two months. We sold meat but skins and camuses were taken by slaughterhouses for nothing. And if you wanted to take them back, you had to buy them. Camuses cost 450 rubles in 2020, and 350 rubles in 2019.” Larisa S., another reindeer herder, was unsatisfied with her dependence on slaughterhouse rules: “You bring a reindeer, they slaughter it and pay only for the meat. If you want to take the camus, then the meat will be cheaper; it will be accepted as of the second sort, and the second sort is phenous meat. That is, we have to sell reindeer and meat according to their terms and conditions.” Slaughterhouses also dropped their prices for meat. In 2020, the average price of venison varied at the slaughterhouses in YNAO from 180 rubles for the second sort to up to 450 rubles for the first sort per kilogram. The reindeer herder Mikhail V. mentioned that the prices had not changed a lot during the past two years: “This year, slaughterhouses took meat for 450 rubles per kg, and the second sort of meat was taken for nothing—180 rubles—and they paid the money much later. In 2018, meat of the first sort was 220 rubles per kg and the second sort of meat cost 140 rubles.” However, reindeer herders had to accept all the terms and conditions imposed by slaughterhouses because, in general, it was more profitable to slaughter a large number of reindeer, especially during the winter high slaughter season.

Due to the COVID-19 pandemic, delayed vaccination of reindeer, limited access to the slaughtery at the state farms (sovkhoz) along with complicated bureaucratic procedures and long waiting times for payment (up to six months), the sovkhoz was less attractive for individual reindeer herders (only 9% of our respondents ever sold their products to state farms). Elena O., a reindeer herder, explained: *“While slaughtering reindeer at the state farm, there was no need to buy the camuses of our reindeer: the farm just slaughtered reindeer, took the carcasses, and gave us back the other parts. However, now it is more difficult to sell reindeer to a state farm: there are a lot of papers to fill out and we have to wait for our money for six months.”*

Regarding prices, trading posts (faktoria) and local merchants offered half the price for reindeer meat. For example, Venera S. noticed: *“This year, the trading post bought reindeer meat for very cheap”.* The reindeer herder Iakov S. mentioned that the COVID-19 pandemic had dropped prices for velvet antlers: *“This year, due to the coronavirus in the tundra, merchants bought the velvet antlers of the first sort for 1000 rubles per kg, the second sort for 800 rubles, and the third for 600 rubles.”* Venera S. compared current prices for velvet antlers with the previous years: “Last year, the price reached 2,700 rubles.” However, collaboration with local merchants who arrived directly in the tundra was more convenient for reindeer herders and helped solve some COVID-19 related issues with supplies. Reindeer herders accepted the exchange of reindeer products for other goods (fuel, food, clothes, nets, spare parts for snowmobiles, diesel generators, pampers for babies, etc.), and they considered these sales as fair and more convenient, especially during the pandemic period, because they did not slaughter lots of reindeer (usually up to five for sustenance needs, i.e., food and making clothes) and could avoid production losses and the logistical costs associated with going to settlements or cities. The reindeer herder Nikolai S. mentioned: “Antipayuta was not on the way, and we had to nomad with the reindeer herd specifically to the slaughterhouse. This was bad because of weight loss by the reindeer. And it also depended on weather conditions—a lot of snow and ice.” Thus, the COVID-19 pandemic could have an impact on decreasing the number of slaughtered reindeer during the winter due to unfair prices and could create delays until a later period. This could lead to a decline in profit and quality of life, especially for poor individual reindeer herders who are supported by the local government. In 2000, a member of a nomadic family earned 5000 rubles per month. 

When slaughtered, YNAO reindeer products are exported by brokers to both primary and value added processors that process, manufacture, and market reindeer products, which are in high demand in the meat, pharmaceutical and beauty industries. However, processors are limited in the number of reindeer products they can process due to gaps in slaughter procedures. First, the quality of products depends on freezing facilities. If the rules of storage are violated, deterioration takes place several times faster [50,51]. Secondly, slaughterhouses are mostly focused on venison, and most other parts of the reindeer are thrown away or delivered as by-products of the third category. Due to the winter slaughter, it is almost impossible for industrial processors to get reindeer fat, reindeer skin and camuses of high quality. The collection of blood is a very complicated procedure that is implemented in YNAO only at state farms due to high technological requirements. This approach of utilizing meat differs completely from the Indigenous peoples’ consumption of reindeer. Of those interviewed, 98% agreed that they use all parts of a reindeer for food and making clothes, shoes, tools, household items, handicrafts, etc. Andrei A. explained: “We eat the whole reindeer, and we eat it raw, sun-dried, boiled, or fried. My family likes hard boiled reindeer head with hoofs. It’s very delicious. Only the reindeer skull is left. Our dogs eat the same food as we do.” Based on the interviews, the ways of utilizing reindeer by reindeer herders and other stakeholders who process reindeer products are presented in Table 3.

The distributors, including wholesalers, market and sell reindeer products in YNAO, and export them to other regions of Russia and abroad. YNAO is the only region in Russia that officially exports reindeer products to the EU. In 2008, four tons of reindeer products from YNAO were exported to Sweden, Finland and Germany [27]. In 2019, the total export of YNAO reindeer products brought in an income of $29 million. A total of 440 tons (24%) of slaughtered reindeer meat and 11,000 reindeer skins from YNAO were exported to Finland and Germany [47]. Now, only three enterprises in YNAO—municipal enterprise Yamal’skie Oleni, municipal unitary enterprise Meat Processing Complex Payuta and LLC Vozrozhdenie—have certification as official exporters of reindeer meat and one more company—LLC Sibirsky olen—specializes in exporting reindeer skins and other by-products to the EU. In 2020, according to the national project “International Cooperation and Export,” 400 tons of reindeer meat and 13,000 reindeer skins are planned to be exported to European countries [27]. Already this year, 270 tons of reindeer meat and 15 tons of other by-products of the third category have been exported to Finland and Germany [27], representing 50% of planned exports. By 2024, the government of YNAO plans to double exported reindeer products and to increase the number of consuming countries. Prospective reindeer products to be exported are bones and blood enriched with collagen, calcium, iron and protein. The COVID-19 pandemic has decreased the export of reindeer products. For example, Finland cancelled the import of 13,000 reindeer skins in March [27]. Tourists are the main consumers of the handicrafts made of skin, and the crisis in the tourism industry has resulted in a decline of demand for these products.

The COVID-19 pandemic has accentuated consumers’ motivation “to protect themselves and their immune system by adopting healthier diets. The availability of bioactive ingredients of food and functional foods may become critical, as the demand for these products may increase” [52]. Due to the increasing export potential of reindeer products, local consumers (both Indigenous and non-Indigenous communities in YNAO) have low access to reindeer meat. Of the interviewed reindeer herders, 83% confirmed that they supply reindeer products to their relatives when they visit them, mostly during the winter period. A further 45% of interviewed individual reindeer herders sold some reindeer meat to local communities in the large settlements. According to official data from the Department of Agro-industrial Complex of YNAO, only 6% of all reindeer were sold to the local population: 2622 reindeer by agricultural enterprises and 2288 by national communities [27]. This has accentuated the trend of a dramatic decline of access to reindeer meat in the local communities. According to monitoring data received by the Arctic Research Scientific Centre, in 2012–2017 20% of the total volume of reindeer meat was consumed by the herders themselves, 18% was sold in Indigenous villages or transferred to relatives and 62% was exported outside the Yamal-Nenets Autonomous Okrug. On average, a family of nomadic Nenets reindeer herders consisting of two to four adults and three to seven children consumed no more than 10–12 reindeer per year. In addition, part of the meat was transported to relatives living in villages with an Indigenous population. A significant portion of reindeer meat was also sold in villages and towns with an Indigenous population or cities close to the herding routes [53].

The COVID-19 pandemic almost completely limited the access of local communities to reindeer products; only 24% of the respondents could supply their relatives with reindeer meat in the settlements. This is because of food safety requirements as “a significant issue in order to avoid the spreading of the virus between producers, retailers, and consumers” [52]. People living in the remote territories of YNAO also had low access to reindeer products. The results of previous research also showed a dramatic decline of almost 50% in consumption of reindeer products by the Indigenous and non-Indigenous peoples in YNAO, and only one third of the studied population still eat venison once or twice daily [54]. This threatens the maintenance of their health since a diet enriched with venison significantly increases antiatherogenic blood lipid fractions, contributes to the maintenance of normal body weight, and improves microcirculation, tissue fluid exchange and antioxidant defense of the body against free radicals, which may explain the high prophylactic activity of venison [54] that has also shown a high efficiency in helping to adapt to cold stress [55] and geomagnetic activity in the Arctic [56]. It can be used as an effective remedy for reducing hypertension [10] and chronic nonobstructive bronchitis risk [13]. This makes reindeer products an important part of the local population’s nutrition.

This systematic research into the reindeer food value chain in YNAO has revealed strong dependence of reindeer herders on other stakeholders—brokers, processors and government institutions,—which makes them a vulnerable group and establishes barriers to entering the global market. Individual reindeer herders are not involved in the complete processing of reindeer products and have to accept the rules imposed by wholesalers and other retailers. However, reindeer herders are keepers of precious traditional knowledge about utilizing reindeer. Strengthening the involvement of reindeer herders in the food value chain can contribute to Arctic Indigenous economies and maintain their traditional lifestyle.

The main strength of our study was using the unique data of quantitative and qualitative research collected from the reindeer herders and local authorities during expeditions that took place over seven years (2014–2020). Most similar studies remain fragmentary, and often hard to access. However, our study had several limitations. The studied population was recruited while undergoing a medical examination at health care institutions—municipal hospitals and feldsher-midwife medical stations in remote settlements. Participation was voluntary and did not include all representatives of the reindeer herding business (i.e., employees of the state farms and commercial agricultural enterprises) of the studied territories, which may limit the generalizability of findings. Future research could also benefit from exploring food security in reindeer herding, on utilizing reindeer in commodity production and on the impact of traditional reindeer products on the health and wellbeing of the local communities.

## 4. Conclusions

In the Arctic zone of Western Siberia, short- and long-term measures are needed to maintain a healthy lifestyle replete with traditional food as part of Indigenous peoples’ food system. The economic position of reindeer herders in the food value chain, who are very dependent on other stakeholders, should be strengthened. This currently creates barriers for integration of reindeer herders into the global market and makes their positions insecure, and the situation is exacerbated by the COVID-19 pandemic. The strengthening the food sovereignty of reindeer herders (i.e., the improvement of their sovereignty over the use, sale and consumption of food) could be reached by improved economic sovereignty. This is also a key factor in solving food security for the local communities since this would mitigate the compulsive export sale of meat to obtain economic resources. Thus, this would improve the food security of the local communities while providing economic security for reindeer herders. Solutions could derive from an equilibrium between regulating and improving private business, state intervention, and cooperative mobilization of reindeer herders or local residents. State intervention is more feasible in some areas, bottom-up mobilization in other areas, while the maintenance of incentives of local private businesses need to be kept in mind. Apart from strengthening economic and food security for reindeer herders it is equally important to strengthen both traditional households and local businesses since they are central to improve the resilience of their communities facing economic, climate and pandemic challenges.

We propose the following:

Short-term measures:
  - Organize a mobile economic and legal consulting service for nomadic and semi-nomadic reindeer herders to support business and establish cooperative reindeer husbandries.
  - Encourage cooperative forms of reindeer husbandry and implement a new government program subsidizing peasant farms for purchase of slaughter, freezing and storage facilities (managed by reindeer herders’ wives and elderly family members staying at the settlements with contribution the Indigenous and non-Indigenous people from the local communities) to contribute to reindeer herding as a traditional family business and increase year-round access of remote local communities to reindeer products.
  - Organize veterinary facilities in the settlements and provide reindeer herders with medications for reindeer.
  - Increase access of reindeer herders to reasonably priced fuel and basic food products near the settlements and trading spots.
  - Organize mobile slaughter and supplemental reindeer feeding facilities in the tundra along the nomadic routes of reindeer herders.
  - Extend the export potential of non-edible parts of reindeer (i.e., velvet antlers, reindeer skins, camuses) to support food sovereignty of the Indigenous peoples, while focusing government policies on improving access of the Indigenous communities to the edible and medicinal portions of the carcass.
Long-term measures
  - Train Indigenous peoples to harvest and process reindeer products according to bioproduction standards and increase their involvement in the complete food value chain as producers, brokers, processors and distributors to strengthen food sovereignty among the Indigenous peoples.
  - Subsidize medications for reindeer and fuel for reindeer herders and develop infrastructure to facilitate the economic empowerment of reindeer herders.
  - Monitor the consumption of traditional food, as well as the health and social welfare of the Indigenous population in the Russian Arctic. Explore ways to maintain traditional nutrition in the local communities.

## Figures and Tables

**Table 1:** Food value chain of reindeer herding products in the Yamal-Nenets Autonomous Okrug (YNAO).

**Stakeholder**	**1. Producers**	**2. “Brokers”**	**3. Processors**	**4. Distributors**	**5. Consumers**
	Agricultural reindeer herding enterprises, peasantry farms, “national communities” (obschina), individual reindeer herders (families)	Gross purchasers (slaughterhouses, agricultural reindeer herding enterprises), trade points (faktoria), local merchants	Companies processing meat products and other by-products in YNAO, Russian and international producers	Export companies, shops, local merchants	Local Indigenous communities, other consumers
Role	Reindeer herding, trading to exporters and local communities	Purchase of reindeer products, storage, veterinary and sanitary control, transportation and delivery to processors or local consumers	Processing, value added processing, manufacturing, marketing and sales	Supplies to the consumer market	Shopping, consuming
Key issues	Dependence on “brokers” requirements and regulations, - high production costs due to insufficient logistics, deficit and remoteness of petroleum stations; low motivation towards business cooperation	High logistic costs; lack of innovative equipment for improved slaughter procedures	Low access to precious reindeer products of high quality (fat, skin and other by-products)	Low share of reindeer products distributed to the local market of YNAO	Low access to reindeer products for the Indigenous Peoples living in remote settlements of YNAO
	**6. Government/NGOs/Regulators**				
	Department of Agro-industrial Complex of YNAO				
	Department of Natural Resources Regulation, Forest Relations and Development of the Oil and Gas Complex of the Yamal-Nenets Autonomous Okrug				
	Department of Indigenous Minorities of the North of the Yamal-Nenets Autonomous Okrug				
	Veterinary Service of the Yamal-Nenets Autonomous Okrug				
	Associations of the Indigenous Peoples of YNAO etc.				
	Role: Public policy on food security and support of reindeer herders				
Key issues	Limited access to subsidies for individual reindeer herders,				
	insufficient legal and economic information support for reindeer herders and insufficient supplies of medications for reindeer,				
	export-oriented economy of reindeer products in YNAO				

**Table 2:** Agricultural enterprises, national communities, peasantry farms and individual reindeer herders in YNAO* (on 1 January 2020).

District of YNAO	Agricultural Enterprises		National Communities of the Indigenous Peoples		Peasantry Farms		Individual Reindeer Herders	
	n	Reindeer	n	Reindeer	n	Reindeer	n	Reindeer
Salekhard	1	1200	1	580	0	0	0	0
Nadymsky	1	14,007	1	292	0	0	92	10,692
Purovsky	2	15,903	0	0	1	1710	137	14,052
Tazovsky	3	20,426	3	16,152	3	2730	1049	215,088
Shuryshkarsky	2	13,532	0	0	1	20	57	7682
Krasnosel’kupsky	2	1021	0	0	0	0	28	644
Yamalsky	6	25,756	436	88,526	3	14,033	761	97,142
Priural’sky	2	14,025	6	28,018	3	5062	715	47,957
Total	19	105,870	447	133,568	11	23,555	2839	393,257

* The data were received from the specialists of the Department of Agro-industrial Complex of YNAO [27] and collected from the Register of the main producers of the Yamal-Nenets Autonomous Okrug [28].

**Table 3:** The utilization of the reindeer products of YNAO.

Product	Average Yield per one Reindeer		Utilization	
	Weight, kg	Yield, %	By Reindeer Herders	By Processors
Total reindeer	66.00	100.00		
Venison with carcass	33.00	50.00		
muscles	20.13	30.50	Raw, sun-dried, frozen (“stroganina”), boiled, fried meat—for food	Meat industry (canned, smoked, salted venison, sausages, chips)
fat	2.48	3.75	Raw—for remedies	Pharmaceutical industry (biologically active medications)
bones	6.93	10.50	Boiled—for food and making tools and household items	Animal feeding stuff
tendons and fascia	3.47	5.25		
Tongue	0.33	0.50	Boiled—for food	Meat industry
Blood	4.22	6.40	Raw—for food and remedies	Pharmaceutical industry (biologically active medications)
Heart	0.42	0.64	Raw and boiled—for food	Meat industry; animal feeding stuff (produced premium hypoallergenic dog and cat food)
Lungs	0.67	1.02		
Liver	0.70	1.06		
Kidneys	0.15	0.22		
Stomach:				
farding bag	3.06	4.64	For keeping fresh blood	Meat industry
honeycomb bag	0.37	0.56	Boiled—for food	
bible-bag	0.55	0.84		
rennet bag	0.36	0.54		
Intestine and esophagus	2.30	3.48		Animal feeding stuff
Gullet and larynx	0.18	0.28		
Antlers	1.06	1.60	Handicrafts, remedy, making tools and household items	Pharmaceutical industry (biologically active medications); beauty industry (cosmetic production enriched with collagen); handicrafts
Hoof	1.08	1.64	Boiled—for food	Beauty industry (cosmetic production enriched with collagen); animal feeding stuff
Skin, camus, forehead	6.97	10.56	Summer skin—for winter “chum” cover, coverage of the floor and “beds”, making clothes, shoes handicrafts	Making clothes, shoes and handicrafts
Tail	0.12	0.18		
Cranial muscles and bones	3.30	5.00	Boiled—for food	Animal feeding stuff
Endocrine, enzymatic and special raw products	2.39	3.62	Raw and boiled—for food	Animal feeding stuff
Wastes (gastric and intestine contents etc.)	4.77	7.22	Boiled—for food	Animal feeding stuff
